# Single Nucleotide Polymorphisms in the *PRDX3* and *RPS19* and Risk of HPV Persistence and Cervical Precancer/Cancer

**DOI:** 10.1371/journal.pone.0033619

**Published:** 2012-04-09

**Authors:** Mahboobeh Safaeian, Allan Hildesheim, Paula Gonzalez, Kai Yu, Carolina Porras, Qizhai Li, Ana Cecilia Rodriguez, Mark E. Sherman, Mark Schiffman, Sholom Wacholder, Robert Burk, Rolando Herrero, Laurie Burdette, Stephen J. Chanock, Sophia S. Wang

**Affiliations:** 1 Division of Cancer Epidemiology and Genetics, National Cancer Institute, Bethesda, Maryland, United States of America; 2 Proyecto Epidemiológico Guanacaste, Fundación INCIENSA, San José, Costa Rica; 3 Academy of Mathematics and Systems Science, Chinese Academy of Sciences, Beijing, China; 4 Albert Einstein College of Medicine, Bronx, New York, United States of America; 5 Core Genotyping Facility, Division of Cancer Epidemiology and Genetics, Advanced Technology Program, SAIC Frederick Inc, NCI-Frederick, Frederick, Maryland, United States of America; 6 Division of Etiology, Department of Population Sciences, City of Hope, Duarte, California, United States of America; University of Navarra, Spain

## Abstract

**Background:**

Host genetic factors might affect the risk of progression from infection with carcinogenic human papillomavirus (HPV), the etiologic agent for cervical cancer, to persistent HPV infection, and hence to cervical precancer and cancer.

**Methodology/Principal Findings:**

We assessed 18,310 tag single nucleotide polymorphisms (SNPs) from 1113 genes in 416 cervical intraepithelial neoplasia 3 (CIN3)/cancer cases, 356 women with persistent carcinogenic HPV infection (median persistence of 25 months) and 425 randomly selected women (non-cases and non-HPV persistent) from the 10,049 women from the Guanacaste, Costa Rica HPV natural history cohort. For gene and SNP associations, we computed age-adjusted odds ratio and p-trend. Three comparisons were made: 1) association with CIN3/cancer (compared CIN3/cancer cases to random controls), 2) association with persistence (compared HPV persistence to random controls), and 3) progression (compared CIN3/cancers with HPV-persistent group). Regions statistically significantly associated with CIN3/cancer included genes for peroxiredoxin 3 *PRDX3*, and ribosomal protein S19 *RPS19*. The single most significant SNPs from each gene associated with CIN3/cancer were *PRDX3* rs7082598 (*P*_trend_<0.0001), and *RPS19* rs2305809 (*P*_trend_=0.0007), respectively. Both SNPs were also associated with progression.

**Conclusions/Significance:**

These data suggest involvement of two genes, *RSP19* and *PRDX3*, or other SNPs in linkage disequilibrium, with cervical cancer risk. Further investigation showed that they may be involved in both the persistence and progression transition stages. Our results require replication but, if true, suggest a role for ribosomal dysfunction, mitochondrial processes, and/or oxidative stress, or other unknown function of these genes in cervical carcinogenesis.

## Introduction

While it is well-known that carcinogenic human papillomaviruses (HPVs) are the causal agents of cervical cancer, HPV infections are extremely common relative to rare cancer incidence, indicating that many infections spontaneously resolve [1], or persist without progression. Host genetic factors may play a role in cervical carcinogenesis and are thought to influence who develops persistent HPV infection and perhaps who further progresses to cancer [2]–[7].

The role of host genetic factors and other co-factors associated with cervical cancer are particularly interesting because the stepwise pathogenesis of the disease has been extensively studied. From its initiation through HPV infection at the cervical transformation zone, and subsequent steps related to viral persistence, progression to precancer, and invasion [1], the same or different factors can be associated with each step towards pathogenesis. The role of non-genetic co-factors in persistence and progression has been well-studied, but there are fewer studies on the host genetics role on the pathogenesis of cervical cancer. Thus, genetic studies of cervical cancer supplement comparisons of cancer cases to non-cancer or uninfected controls, by investigating each intermediate causal step, namely persistent infection and progression to CIN3/cancer.

We used data from the well-characterized, longitudinal cohort study on HPV natural history (NHS) in Guanacaste, Costa Rica, and recently reported results from a panel of 7,140 candidate single nucleotide polymorphisms (SNPs). These polymorphisms were chosen to represent variation in 305 genes based on *a priori* hypotheses of association with HPV infection and cervical cancer (DNA repair, viral infection and cell entry pathways). That effort identified 8 potential genes associated with cervical cancer, including the immune genes 2′,5′ oligoadenylate synthetase gene 3 (*OAS3*) and sulfatase 1 (*SULF1*), and the epidermal dysplasia verruciformis (EV)-associated *EVER1* and *EVER2* genes, *TMC6* and *TMC8* [*8*]. We now report our findings regarding the remaining 18,310 SNPs (covering 1,113 genes) that were genotyped on the same iSelect chip. These additional SNPs were selected based on their *a priori* hypothesized relationship with a wide range of cancers, but not specifically with HPV persistence and cervical cancer. These genes were selected based on the collective effort of numerous cancer researchers and include genes in several immune, cytokine and inflammation response, DNA replication and differentiation, macrophage differentiation, toll-like receptor signaling, and T cell receptor signaling pathways, to name a few, and presumably have lower prior probabilities of association with cervical cancer than those studied in the report of Wang et al. [8].

## Results

### Gene region-based associations

Table 1 shows results for 14 genes/regions with p<0.005 for association with either disease (CIN3/cancer versus controls), disease progression (CIN3/cancer versus HPV persistence), or persistent infection (HPV persistence versus controls) (arranged by p-value for disease associations). This analysis identified 9 gene regions as statistically significantly associated with CIN3/cancer at a p-value of ≤0.005 (*PRDX3* p-value 0.00015; *RPS1*9 p-value 0.00045; *DDX1* p-value 0.0006; *TELO2* p-value 0.0009; *C1RL* p-value 0.00165; *ILDR1* p-value 0.00285; *THRAP4* p-value 0.0037; *GDF10* p-value 0.004; and *GDF2* p-value 0.004). Two gene regions were identified as statistically significantly associated with disease progression at p-value of ≤0.005 (*GC* p-value 0.0004; and *IL2RA* p-value 0.00115). In addition, 2 gene regions were identified as significantly associated with type-specific HPV persistence (*TYMS* p-value 0.0015; and *EVPL* p-value 0.0018). Of the genes identified to be associated with CIN3/cancer *RPS19* was also associated with progression to CIN3/cancer (p-value 0.006). Similarly, of the genes associated with CIN3/cancer, *C1RL*, *GDF10*, and *GDF2* were also associated with persistence (p-values of 0.00875, 0.0423, and 0.04080, respectively). Only two of the genes - *PRDX3* and *RPS19*, were notable with a FDR≤0.2 comparing CIN3/cancers to random controls. All gene-based results are shown in Table S1.

**Table 1:** Significance levels (p values) for gene regions with p≤0.005, for either an association with (i) cervical precancer/cancer, (ii) progression to cervical precancer/cancer, (iii) HPV persistence.

	Chromosome	# SNPs	(i)	(ii)	(iii)
Gene			CIN3+ (n=415)	CIN3+ (n=415)	HPV persistence (n=356)
			vs. RC (n=425)†	vs. HPV persistence (n=356)	vs. RC (n=425)
*PRDX3* *	10q25-q26	11	**0.00015**	0.0744	0.16314
*RPS19* *	19q13.2	4	**0.00045**	0.00585	0.45823
*DDX1*	2p24	13	**0.0006**	0.69087	0.21284
*TELO2*	16p13.3	20	**0.0009**	0.17849	0.65932
*C1RL*	12p13.31	20	**0.00165**	0.14029	0.00875
*ILDR1*	3q13.33	11	**0.00285**	0.05830	0.88461
*THRAP4*		6	**0.00370**	0.80776	0.05615
*GDF10*	10q11.22	20	**0.00400**	0.13639	0.04230
*GDF2*	10q11.22	19	**0.00400**	0.12639	0.04080
*TYMS*	18p11.32	20	0.0055	0.30233	**0.0015**
*EVPL*	17q25	10	0.02055	0.08985	**0.00180**
*GC*	4q12-q13	19	0.0343	**0.0004**	0.00965
*IL2RA*	10p15-p14	44	0.24394	**0.00115**	0.44318
*PIK3CA*	3q26.3	11	0.91060	0.05145	**0.00490**

* FDR for both PRDX3 and RPS19=0.19.

† RC=random controls.

### SNP-based associations

Six SNPs in 6 genes identified by the gene-based analysis were significantly associated with CIN3/cancer, disease progression, or persistent oncogenic HPV infection (Table 2) in SNP-based analysis. The *PRDX3* rs7082598 minor allele variants was associated with statistically significant decreased risk of cervical cancer compared to controls (OR_CT/TT_=0.41, 95%CI 0.30–0.58, *P*_trend_<0.0001), and this protective effect was observed for both progression (CIN3+ compared with HPV-persistence) and HPV-persistence (persisters compared to controls) (OR_CT/TT_=0.58, 95%CI 0.40–0.83 *P*_trend_=0.008, and 0.71, 95%CI 0.52–0.97, *P*_trend_=0.02 respectively). The *RPS19* rs2305809 T allele was also associated with statistically significant decreased risk of cervical cancer (OR_CT_=0.66, 95%CI 0.48–0.91; OR_TT_=0.43, 95%CI 0.28–0.66, *P*_trend_=0.00007). Further examination by intermediate steps showed that this effect remained for progression from persistence to CIN3+ (OR_CT_=0.83, 95%CI 0.59–1.15; OR_TT_=0.51, 95%CI 0.33–0.78, *P*_trend_=0.003). Lastly, the *IL2RA* rs2476491 T variant was also associated with significantly decreased risk of cervical cancer (OR_AT/TT_=0.69, 95%CI 0.51–0.92, *P*_trend_=0.02). This association was strongest for progression from HPV-persistence to CIN3+ (OR_AT/TT_=0.53, 95%CI 0.39–0.71, *P*_trend_=0.00002).

**Table 2:** Odds ratios and 95% confidence intervals for top-ranked SNPs (p≤0.0001) with either an association with (i) cervical precancer/cancer, (ii) progression to cervical precancer/cancer, or (iii) HPV persistence: (all models adjusted for age).

Gene	rs #	Random control	HPV persistence	CIN3/cancer	CIN3/cancer	CIN3/cancer vs	HPV persistence
					vs RC	HPV persistence	vs RC
		N (%)	N (%)	N (%)	OR (95% CI)	OR (95% CI)	OR (95% CI)
***PRDX3***	**RS7082598**						
	**CC**	288 (68)	266 (75)	346 (83)	1.0 (ref)	1.0 (ref)	1.0 (ref)
	**CT**	127 (30)	87 (24)	64 (15)	0.41 (0.29–0.58)	0.55 (0.38–0.80)	0.74 (0.53–1.01)
	**TT**	10 (2)	3 (1)	5 (1)	0.45 (0.15–1.35)	1.29 (0.29–5.65)	0.33 (0.09–1.20)
					p-trend<0.00001	p-trend=0.008	p-trend=0.02
	**CT/TT**	137 (32)	90 (25)	69 (17)	0.41 (0.30–0.58)	0.58 (0.40–0.83)	0.71 (0.52–0.97)
***RPS19***	**RS2305809**						
	**CC**	100 (24)	102 (29)	141 (34)	1.0 (ref)	1.0 (ref)	1.0 (ref)
	**CT**	228 (54)	172 (48)	213 (51)	0.66 (0.48–0.91)	0.83 (0.59–1.15)	0.74 (0.53–1.05)
	**TT**	97 (23)	82 (23)	60 (14)	0.43 (0.28–0.66)	0.51 (0.33–0.78)	0.83 (0.56–1.25)
					p-trend=0.00007	p-trend=0.003	p-trend=0.34
	**CT/TT**	325 (76)	254 (71)	273 (66)	0.59 (0.43–0.80)	0.72 (0.53–0.99)	0.77 (0.56–1.06)
***Il2RA***	**RS2476491**						
	**AA**	262 (62)	196 (55)	289 (70)	1.0 (ref)	1.0 (ref)	1.0 (ref)
	**AT**	147 (35)	135 (38)	114 (27)	0.69 (0.51–0.93)	0.56 (0.41–0.77)	1.22 (0.91–1.65)
	**TT**	15 (4)	25 (7)	12 (3)	0.68 (0.31–1.5)	0.34 (0.16–0.70)	2.24 (1.15–4.37)
					p-trend=0.02	p-trend=0.00002	p-trend=0.02
	**AT/TT**	162 (38)	160 (45)	126 (30)	0.69 (0.51–0.92)	0.53 (0.39–0.71)	1.32 (0.99–1.76)
***TELO2***	**RS4786772**						
	**AA**	185 (44)	151 (42)	134 (32)	1.0 (ref)	1.0 (ref)	1.0 (ref)
	**AG**	197 (46)	147 (41)	211 (51)	1.54 (1.14–2.09)	1.65 (1.19–2.27)	0.92 (0.68–1.24)
	**GG**	42 (10)	58 (16)	69 (17)	2.51 (1.59–3.94)	1.40 (0.91–2.15)	1.68 (1.06–2.65)
					p-trend=0.00002	p-trend=0.03	p-trend=0.13
	**AG/GG**	239 (56)	205 (58)	280 (68)	1.71 (1.28–2.28)	1.57 (1.16–2.13)	1.05 (0.79–1.39)
***C1RL***	**RS12227050**						
	**GG**	402 (95)	310 (87)	357 (86)	1.0 (ref)	1.0 (ref)	1.0 (ref)
	**AG**	22 (5)	45 (13)	57 (14)	2.91 (1.73–4.89)	1.06 (0.69–1.62)	2.73 (1.60–4.65)
	**AA**	1 (0.2)	1 (0.3)	1 (0.2)	1.37 (0.08–22.18)	0.77 (0.05–12.91)	1.36 (0.08–21.92)
					p-trend=0.0001	p-trend=0.85	p-trend=0.0004
	**AG/AA**	23 (5)	46 (13)	58 (14)	2.85 (1.71–4.74)	1.05 (0.69–1.61)	2.67 (1.58–4.51)
***TYMS***	**RS2342700**						
	**CC**	227 (53)	145 (41)	169 (41)	1.0 (ref)	1.0 (ref)	1.0 (ref)
	**CG**	169 (40)	164 (46)	199 (48)	1.56 (1.17–2.09)	1.06 (0.78–1.44)	1.53 (1.13–2.06)
	**GG**	29 (7)	47 (13)	46 (11)	2.18 (1.30–3.64)	0.87 (0.54–1.40)	2.57 (1.54–4.27)
					p-trend=0.0002	p-trend=0.78	p-trend=0.00005
	**CG/GG**	198 (47)	211 (59)	245 (59)	1.65 (1.25–2.18)	1.02 (0.76–1.37)	1.68 (1.26–2.23)

Variants alleles in *TELO2* rs4786772 (OR_AG_=1.54, 95%CI 1.14–2.09; OR_GG_=2.51, 95%CI 1.59-3.94, *P*_trend_=0.00002), *C1RL* rs12227050 (OR_AG/GG_=2.85, 95%CI 1.71–4.74, *P*_trend_=0.0001), and *TYMS* rs2342700 (OR_CG_=1.56, 95%CI 1.17–2.09; OR_GG_=2.18, 95%CI 1.30–3.64, *P*_trend_=0.0002) were associated with significantly increased risk of cervical cancer (Table 2). The increased risk of variants for *TELO2* remained for progression from persistence to CIN3+, while the increased risk for *C1RL* and *TYMS* remained for persistence compared to random controls.

We also carried out a single statistical analysis including all SNPs, and found that the SNP RS7082598 (*PRDX3* gene) retained significance in this analysis when comparing CIN3+ vs. random controls (multiple comparison adjusted p-value 0.011).

## Discussion

In this analysis of genes and SNPs identified to be broadly relevant for cancer etiology, but not specifically for cervical carcinogenesis, we found 14 genes/regions that were significantly associated with CIN3/cancer at p<.005. Two of these genes – *RPS19* and *PRDX3* – were notable at a FDR≤0.2. Replication of these results is warranted to eliminate the role of chance finding.

Effects of putative genetic etiologic factors for cervical cancer may be mapped to specific transition states from HPV infection of the cervical transformation zone, progression of persistently infected cervical cells to precancer and invasive cancer. In this study we had the opportunity to investigate and identify genes which may influence some of these stages of cervical carcinogenesis. We note that the protective effects observed for *PRDX3* and *RPS19* SNPs remained for both disease progression (from HPV-persistent infection to CIN3+) and for HPV-persistence.

Mutations of the ribosomal gene *RPS19* have been associated with Diamond-Blackfan anemia (DBA), which is a constitutional erythroblastopenia characterized by absent or decreased erythroid precursors, in a subset of patients. Patients with DBA have increased risk of osteosarcoma. This association with DBA suggests a possible extra-ribosomal function for this gene in erythropoietic differentiation and proliferation, in addition to its ribosomal function. In some primary colon carcinomas, higher expression levels of this gene have been observed compared to matched normal colon tissues [9]. *PRDX3* is in the peroxiredoxin family which encodes a protein with antioxidant function, is localized in the mitochondrion, and may function to protect mitochondria from oxidative stress. Sequence comparisons with recently cloned mammalian homologues suggest that these genes consist of a family that is responsible for regulation of cellular proliferation, differentiation, and antioxidant functions [10], [11]. Neither of these genes has an obvious relationship to the known carcinogenic processes that lead to cervical cancer.

There are several study limitations to be considered. We combined the CIN3+ cases from the NHS cohort-based study with the CIN3+ supplemental cases drawn from the community at the same period for increased analytic power. There was a higher proportion of CIN3 (a corollary of detection by screening in NHS rather than symptoms) in the NHS cases than supplemental cases; additionally supplemental cases were older, mainly because of the larger proportion of cancers compared to the NHS cohort-based cases where there were higher proportion of CIN3. Tests of associations between NHS and supplemental populations for 94.2% (17,149/18,208) of the SNPs were not significant at 5% level, importantly, all the SNPs associated with the two genes of interest (*PRDX3* and *RPS19*) were similarly distributed between NHS and supplemental cases, justifying combining the two cohorts. Additionally, this study was powered on a combined end-point of CIN3 and cancer; 95.5% (17,394/18,208) of the SNPs were not statistically different between CIN3 and cancer cases. Importantly, none of SNPs associated with the two genes of interest (*PRDX3* and *RPS19*) were statistically significantly different between CIN3 and cancers. Because SNPs were chosen as tagging markers for genetic regions rather than function, the observed associations with SNPs may be due to linkage disequilibrium with other causal unmeasured SNPs. We were underpowered to perform analyses restricted to carcinogenic HPV types due to small sample size as only women enrolled in the original NHS had HPV typing data. Future studies should therefore consider HPV genotypes in analysis. We were also unable to evaluate genetic factors associated with invasive cervical cancer separately. Future studies with large number of cases will be required to address whether certain genes are associated with transition from in situ to invasive cervical cancer. Although we performed haplotype-based analyses (defined by blocks of linkage disequilibrium); results were generally consistent with the gene region- and SNP-based findings. No new regions of interest were identified in haplotype analysis using the sliding window approach of 3 SNPs. The genes evaluated here were also not selected based on their previously reported associations with cervical cancer, but by agnostic analysis, encompassing a global effort to identify genes involved with a range of infection and non-infection-related cancers.

In summary, these data suggest involvement of two genes, *RSP19* and *PRDX3*, or other SNPs in linkage disequilibrium, with cervical cancer risk. Further investigation showed that they may be involved in both the persistence and progression transition stages. If replicated, these results may suggest a role for ribosomal dysfunction, mitochondrial processes, and/or oxidative stress, or as yet unknown function in cervical cancer pathogenesis.

## Materials and Methods

### Study population

Data are from the Guanacaste HPV Natural History Study (NHS), a population-based cohort study in Guanacaste, Costa Rica. Details of the cohort study methods [12], [13] and of the sub-population selected for genetic analyses [14], [15] have been reported elsewhere. Briefly, NHS is a population-based cohort of 10,049 women recruited over an 18-month period in 1993-4 and followed for seven years. The primary objective of NHS was to study the natural history of HPV infection and cervical intraepithelial neoplasia (CIN). Cervical cells were available for HPV DNA testing, and buffy coat specimens were available for host gene polymorphism studies.

A host genetic sub-study was nested within NHS, as described previously [14], [15]. Briefly, individuals selected for the genetic sub-study included: (i) all women in the cohort histologically confirmed to have prevalent or incident CIN3 or cancer (CIN3+: CIN3=140, cancer=45); (ii) all women in the cohort who at the time of selection into the study had evidence of HPV persistence, defined as women positive for the same HPV type (either carcinogenic or not) at two consecutive visits at least 12 months apart (n=432) (median length of observed persistence: 25 months); and (iii) a random selection of participants without CIN3, or cancer from the baseline cohort (n=492). To increase power for studies of host genetics, we conducted a supplemental substudy that captured all CIN3 (n=240) and cancer cases (n=87) who were not participants in NHS but were independently diagnosed with CIN3 or cancer at Social Security clinics from the same study area and during the same period in which NHS was conducted [15]. The study was approved by both the US NCI and Costa Rica Institutional Review Boards and all subjects signed informed consent.

### Laboratory Methods

#### DNA extraction from blood

DNA was extracted from buffy coats with PureGene purification kits/Autopure protocol (Gentra Systems) at SeraCare (Frederick, MD). For the supplemental cases the DNA extraction was done at the University of Costa Rica using the same kit.

#### HPV testing

PCR-based HPV DNA testing was conducted using the L1 MY09/MY11 consensus primer methods [12], [16], [17] on cervical cells stored in specimen transport media (Qiagen, USA) from the natural history study only. Because cervical cells were not obtained from the supplemental cases, HPV results are restricted to women within the original cohort.

#### Host genotyping

A panel was designed as part of an effort of numerous investigators based on their expertise in specific cancers. Genotyping of tag SNPs from 990 candidate gene regions hypothesized to be involved in wide number of cancers such as colon, osteosarcoma, esophageal and stomach, biliary, Fanconi anemia, bladder, breast and other cancers, was conducted at the NCI Core Genotyping Facility (Advanced Technology Center, Gaithersburg, MD; http://snp500cancer.nci.nih.gov) [18] using a custom-designed iSelect Infinium assay (Illumina, www.illumina.com). The Infinium included a total of 27,904 tag SNPs. Tag SNPs of the genes were chosen from the designable set of common SNPs (minor allele frequency (MAF>5%) genotyped using the all 3 HapMap populations for tagging dependent on the population of interest for the investigator suggesting the candidate gene (Data Release 20/Phase II, NCBI Build 36.1 assembly, dbSNPb126) using the software Tagzilla (http://tagzilla.nci.nih.gov/), which implements a tagging algorithm based on the pairwise binning method of Carlson *et al*. [19]. For each original target gene, SNPs within the region spanning 20 kb 5′ of the start of transcription (exon 1) to 10 kb 3′ of the end of the last exon were grouped using a binning threshold of r^2^>0.8 to define a gene region. When there were multiple transcripts available for genes, only the primary transcript was assessed.

#### Quality control (QC)

Tag SNPs that failed manufacturing (ordered but failed assay development), failed validation (no amplification or clustering) and assays that had less than 80% completion or 80% concordance with the 270 HapMap samples used for validation were excluded (n=269). SNPs with low completion rate (<90% of samples) were further excluded (N=482). SNPs with QC discordance among our 100 QC duplicates and among HapMap samples <98% were excluded (n=1,703). We also excluded samples with a completion rate <90% (n=7). Hardy–Weinberg equilibrium was evaluated among controls, 49 SNPs showed evidence of deviation from Hardy–Weinberg proportions. Our QC data did not suggest any obvious genotyping error in these 49, and their results are therefore presented. Of the 20,764 SNPs, 18,310 SNPs from 1113 genes were included in our present analysis.

#### Final analytic population

We evaluated a total of 416 women diagnosed with CIN3 or cancer, 356 women with HPV persistent infection, and 425 random controls for whom validated genotyping results were obtained.

### Statistical Analysis

#### Gene-based analyses

We obtained a gene-level summary of association using the adaptive combination of p-values [20], which combines gene-level association evidence through adaptive rank truncated product method. We highlight results of the genes that were significant at p-value<0.005. Because some of our results could be due to false-positive findings, we calculated the false discovery rate (FDR) among associations considered significant using the method of Benjamini and Hochberg [21] to the gene region-based tests ^19^. We considered an FDR value of <0.2 as notable.

#### SNP-based associations

We calculated odds ratios (OR) and 95% confidence intervals (95% CI) for each genotype with each disease outcome (CIN3/cancers vs. random controls; HPV-persistence vs. CIN3/cancers; HPV-persistence vs. random controls), using the homozygous wild type (WT) genotype as the referent group. We first compared CIN3/cancer cases to random controls. We further evaluated their associations for HPV progression and/or persistence by comparing: the group of CIN3/cancer cases (n=415) to HPV persisters (n=356) for evaluation of SNPs relevant to progression and (ii) HPV persisters (n=356) to random controls (n=425) for evaluating SNPs relevant to persistence. We note that persistence does not always precede CIN3, as it may be a result of a CIN3 lesion.

We conducted both crude and age-adjusted (<30, 30–49, 50+ years) analyses. For each outcome, we calculated the *P*_trend_ based on the three-level ordinal variable (0, 1, and 2) of homozygote wildtype, heterozygote, and homozygote variant in a logistic regression model. Because of the small cell size examining some of the SNPs (less than 5%), we also show combined effect of heterozygous and homozygous variant genotypes; thus, results of the two-level models are discussed. All logistic regression models were unconditional and conducted using SAS version 9.1 (SAS Institute, Cary, NC).

## Supporting Information

Table S1 Results for all gene based tests for either an association with (i) cervical precancer/cancer, (ii) progression to cervical precancer/cancer, or (iii) HPV persistence.
(XLS)
